# Impact of overnight 1 mg dexamethasone on vascular function in patients with nonfunctioning adrenal adenomas

**DOI:** 10.1038/s41598-023-48295-y

**Published:** 2023-11-28

**Authors:** Shinji Kishimoto, Tatsuya Maruhashi, Masato Kajikawa, Aya Mizobuchi, Takayuki Yamaji, Takahiro Harada, Yukiko Nakano, Chikara Goto, Farina Mohamad Yusoff, Ayumu Nakashima, Yukihito Higashi

**Affiliations:** 1 Department of Regenerative Medicine, Division of Radiation Medical Science, Research Institute for Radiation Biology and Medicine, Hiroshima University, 1-2-3 Kasumi, Minami-Ku, Hiroshima, 734-8551 Japan; 2 Division of Regeneration and Medicine, Medical Center for Translational and Clinical Research, Hiroshima University Hospital, Hiroshima, Japan; 3 Center for Radiation Disaster Medical Science, Research Institute for Radiation Biology and Medicine, Hiroshima University, Hiroshima, Japan; 4 Center for Cause of Death Investigation Research, Graduate School of Biomedical and Health Sciences, Hiroshima University, Hiroshima, Japan; 5 Department of Cardiovascular Medicine, Graduate School of Biomedical and Health Sciences, Hiroshima University, Hiroshima, Japan; 6 Department of Rehabilitation, Faculty of General Rehabilitation, Hiroshima International University, Hiroshima, Japan; 7 Department of Nephrology, Graduate School of Medicine, University of Yamanashi, Yamanashi, Japan

**Keywords:** Cardiology, Endocrinology

## Abstract

The purpose of this study was to evaluate the effects of administration of overnight 1 mg dexamethasone on vascular function in patients with nonfunctioning adrenal adenomas (NFA). Flow-mediated vasodilation (FMD) and nitroglycerine-induced vasodilation (NID) were measured to assess vascular function in 22 patients with NFA who had hypertension and/or diabetes mellitus (DM) and 272 patients without adrenal incidentalomas who had hypertension and/or DM (control patients with hypertension and/or DM). FMD and NID were measured in the morning before and after administration of 1 mg of dexamethasone at 2300 h in 18 patients with NFA. There were no significant differences in FMD and NID between control patients with hypertension and/or DM and patients with NFA who had hypertension and/or DM (3.4 ± 2.8% vs. 2.9 ± 1.9% and 11.5 ± 5.7% vs. 11.4 ± 4.3%, P = 0.46, and P = 0.99, respectively). There were no significant differences in vascular function between control patients with hypertension and/or DM and patients with NFA who had hypertension and/or DM even after adjustment for cardiovascular risk factors. Overnight 1 mg dexamethasone increased FMD from 2.4 ± 1.9% to 5.3 ± 3.2% (P < 0.01) and increased NID from 12.1 ± 4.2% to 14.0 ± 2.8% (P < 0.01) in patients with NFA. The overnight 1 mg dexamethasone suppression test does not impair FMD and NID in patients with NFA. Decreases in circulating levels of cortisol may improve vascular function.

**Clinical Trial Registration:** This study was approved by principal authorities and ethical issues in Japan (URL for Clinical Trial: http://www.umin.ac.jp/ctr/index.htm Registration Number for Clinical Trial: UMIN000039512).

## Introduction

Adrenal incidentalomas are adrenal tumors that are detected unexpectedly during imaging procedures conducted for unrelated reasons. Most of the adrenal incidentalomas are classified as benign nonfunctioning adrenal adenomas (NFA), and patients with NFA which have lower rates of cardiovascular events and mortality than those in patients with autonomous cortisol secretion^1,2^. However, Patrova et al.^3^ showed that mortality of cardiovascular disease in patients with NFA was higher than that in patients without adrenal incidentalomas, while Lopez et al.^4^ showed that the incidence of cardiovascular events in patients with NFA was comparable to that in patients without adrenal adenomas. It is controversial whether patients with NFA have greater risks of cardiovascular events than patients without NFA. It was reported that patients with NFA had an elevated risk of developing hypertension and diabetes mellitus (DM) compared to that in patients without adrenal incidentalomas^4,5^. Androulakis et al.^6^ showed that endothelial function was impaired in NFA patients without hypertension, DM, and/or dyslipidemia compared with that in healthy subjects. However, there is no information on vascular function in patients with NFA who have hypertension and/or DM.

Administration of glucocorticoids for more than three months causes cardiovascular events, even with less than 7.5 mg of prednisolone^7–9^. Administration of cortisol at a dose of 20 mg for five days was also associated with the induction of vascular dysfunction^10^. It is well known that administration of dexamethasone decreases circulating levels of cortisol under the condition of normal adrenal function. The overnight 1 mg dexamethasone suppression test is commonly used diagnostic tool for the purpose of screening individuals for subclinical Cushing’s syndrome. However, the impacts of overnight 1 mg dexamethasone and dexamethasone-induced changes in circulating levels of cortisol on vascular function remain unclear.

Endothelial dysfunction represents an initial stage in the pathogenesis and advancement of atherosclerosis, leading to increased cardiovascular complications^11,12^. Measurements of flow-mediated vasodilation (FMD) and nitroglycerine-induced vasodilation (NID) in the brachial artery are widely recognized as reliable indicators of endothelial function and vascular smooth muscle function, respectively, with significant predictive values for cardiovascular events^13,14^.

The purpose of this study was to evaluate vascular function in patients with NFA who have hypertension and/or DM and the effects of administration of overnight 1 mg dexamethasone on vascular function in those patients.

## Results

### Study protocol 1

#### Baseline clinical characteristics

The baseline clinical characteristics of the 272 control patients with hypertension and/or DM and 22 patients with NFA who had hypertension and/or DM are summarized in Supplemental Table S1. There were significant differences in previous coronary heart disease, use of mineralocorticoid receptor blockers, use of beta-blockers, and any medically treated DM between the two groups.

Moreover, we assessed vascular function in patients with NFA who had hypertension and/or DM using propensity score matching to create matched pairs between control patients with hypertension and/or DM and patients with NFA who had hypertension and/or DM. In propensity score-matched pairs of control patients with hypertension and/or DM and patients with NFA who had hypertension and/or DM, the clinical characteristics of matched pairs of 22 control patients with hypertension and/or DM and 22 patients with NFA who had hypertension and/or DM are summarized in Table 1. There were no significant differences in the baseline characteristics between the two groups.

**Table 1:** Clinical Characteristics of Propensity Score-matched Pairs of Subjects in Protocol 1.

Variables	Control (n = 22)	Nonfunctioning adrenal adenomas (n = 22)	P value
Age, year	65 ± 12	64 ± 10	0.73
Sex, men/women	19/3	18/4	0.68
Body mass index, kg/m^2^	25.8 ± 2.9	24.0 ± 6.8	0.28
Systolic blood pressure, mmHg	134 ± 21	135 ± 17	0.84
Diastolic blood pressure, mmHg	80 ± 12	80 ± 13	0.97
Heart rate, bpm	80 ± 13	76 ± 13	0.97
Total cholesterol, mmol/L	4.60 ± 0.88	4.84 ± 0.75	0.37
Triglycerides, mmol/L	1.47 ± 0.95	1.82 ± 1.47	0.39
High-density lipoprotein cholesterol, mmol/L	1.40 ± 0.36	1.42 ± 0.52	0.85
Low-density lipoprotein cholesterol, mmol/L	2.72 ± 0.65	2.79 ± 0.67	0.68
Glucose, mmol/L	7.44 ± 1.39	6.16 ± 1.22	0.06
Hemoglobin A1c, %	6.1 ± 0.8	6.1 ± 0.8	0.99
Blood urea nitrogen, mmol/L	5.36 ± 1.79	5.36 ± 1.43	0.89
Creatinine, μmol/L	74.26 ± 28.29	73.37 ± 21.22	0.92
Current smoker, n (%)	4 (18.2)	6 (27.3)	0.47
Medical history, n (%)			
Hypertension	19 (86.4)	20 (90.4)	0.63
Dyslipidemia	15 (68.2)	13 (59.1)	0.53
Diabetes mellitus	8 (36.4)	7 (31.8)	0.75
Previous coronary heart disease	0 (0.0)	0 (0.0)	N/A
Previous stroke	2 (9.1)	3 (13.6)	0.63
Medication, n (%)			
Calcium channel blockers	10 (45.5)	12 (54.6)	0.55
Angiotensin-converting enzyme inhibitors	0 (0.0)	0 (0.0)	N/A
Angiotensin II receptor blockers	11 (50.0)	9 (40.9)	0.54
Mineralocorticoid receptor blockers	2 (9.1)	0 (0.0)	0.09
Beta-blockers	3 (13.6)	1 (4.6)	0.28
Alpha-blockers	0 (0.0)	1 (4.6)	0.24
Statins	8 (36.4)	6 (27.3)	0.52
Nitrates	0 (0.0)	0 (0.0)	N/A
Medically treated diabetes mellitus			
Any	4 (18.2)	2 (9.1)	0.37
Insulin-dependent	1 (4.6)	0 (0.0)	0.24
Flow-mediated vasodilation, %	3.5 ± 3.3	2.9 ± 1.9	0.50
Nitroglycerine-induced vasodilation, %	11.0 ± 4.5	11.4 ± 4.3	0.70

N/A indicates not applicable.

Results are presented as means ± SD for continuous variables and percentages for categorical variables.

#### Vascular function in patients without adrenal incidentalomas and patients with NFA

There were no significant differences in FMD and NID between control patients with hypertension and/or DM and patients with NFA who had hypertension and/or DM (3.4 ± 2.8% vs. 2.9 ± 1.9% and 11.5 ± 5.7% vs. 11.4 ± 4.3%, P = 0.46, and P = 0.99, respectively) (Supplemental Table S1).

In propensity score-matched pairs of control patients with hypertension and/or DM and patients with NFA who had hypertension and/or DM, there were no significant differences in FMD and NID between control patients with hypertension and/or DM and patients with NFA who had hypertension and/or DM (3.5 ± 3.3% vs. 2.9 ± 1.9% and 11.0 ± 4.5% vs. 11.4 ± 4.3%, P = 0.50 and P = 0.70, respectively) (Table 1).

### Study protocol 2

#### Baseline clinical characteristics

The baseline clinical characteristics of the 320 control patients and 18 patients with NFA are summarized in Supplemental Table S2. There were significant differences in previous coronary heart disease and any medically treated DM between the two groups.

Moreover, we assessed vascular function in patients with NFA using propensity score matching to create matched pairs between control patients and patients with NFA. In propensity score-matched pairs of control patients and patients with NFA, the clinical characteristics of matched pairs of 18 control patients and 18 patients with NFA are summarized in Table 2. There were no significant differences in the baseline characteristics between the two groups.

**Table 2:** Clinical Characteristics of Propensity Score-matched Pairs of Subjects in Protocol 2.

Variables	Control (n = 18)	Nonfunctioning adrenal adenomas (n = 18)	P value
Age, year	65 ± 14	65 ± 10	0.92
Sex, men/women	13/5	14/4	0.70
Body mass index, kg/m^2^	26.1 ± 4.7	25.8 ± 2.6	0.85
Systolic blood pressure, mmHg	137 ± 15	134 ± 15	0.56
Diastolic blood pressure, mmHg	83 ± 12	79 ± 11	0.37
Heart rate, bpm	71 ± 12	74 ± 12	0.55
Total cholesterol, mmol/L	4.60 ± 1.09	4.84 ± 0.72	0.47
Triglycerides, mmol/L	1.75 ± 1.52	1.93 ± 1.61	0.76
High-density lipoprotein cholesterol, mmol/L	1.42 ± 0.52	1.32 ± 0.49	0.57
Low-density lipoprotein cholesterol, mmol/L	2.64 ± 0.83	2.84 ± 0.62	0.41
Glucose, mmol/L	6.94 ± 1.61	6.11 ± 1.11	0.09
Hemoglobin A1c, %	6.2 ± 0.9	5.9 ± 0.8	0.44
Blood urea nitrogen, mmol/L	5.00 ± 1.43	5.36 ± 1.79	0.43
Creatinine, μmol/L	63.65 ± 13.26	76.02 ± 13.26	0.07
Current smoker, n (%)	1 (5.6)	3 (16.7)	0.28
Medical history, n (%)			
Hypertension	15 (83.3)	15 (83.3)	1.00
Dyslipidemia	11 (61.1)	11 (61.1)	1.00
Diabetes mellitus	4 (22.2)	4 (22.2)	1.00
Previous coronary heart disease	0 (0.0)	0 (0.0)	N/A
Previous stroke	1 (5.6)	3 (16.7)	0.28
Medication, n (%)			
Calcium channel blockers	11 (61.1)	8 (44.4)	0.32
Angiotensin-converting enzyme inhibitors	0 (0.0)	0 (0.0)	1.00
Angiotensin II receptor blockers	3 (16.7)	6 (33.3)	0.24
Mineralocorticoid receptor blockers	0 (0.0)	0 (0.0)	N/A
Beta-blockers	3 (16.7)	1 (5.6)	0.28
Alpha-blockers	0 (0.0)	1 (5.6)	0.23
Statins	6 (27.8)	5 (27.8)	0.72
Nitrates	0 (0.0)	0 (0.0)	N/A
Medically treated diabetes mellitus			
Any	2 (11.1)	1 (5.6)	0.54
Insulin-dependent	0 (0.0)	0 (0.0)	N/A
Flow-mediated vasodilation, %	3.4 ± 2.4	2.4 ± 1.9	0.19
Nitroglycerine-induced vasodilation, %	13.6 ± 4.6	12.1 ± 4.2	0.35

N/A indicates not applicable.

Results are presented as means ± SD for continuous variables and percentages for categorical variables.

#### Impacts of overnight 1 mg dexamethasone suppression test on vascular function and variables in patients with NFA

**Figure 1:**
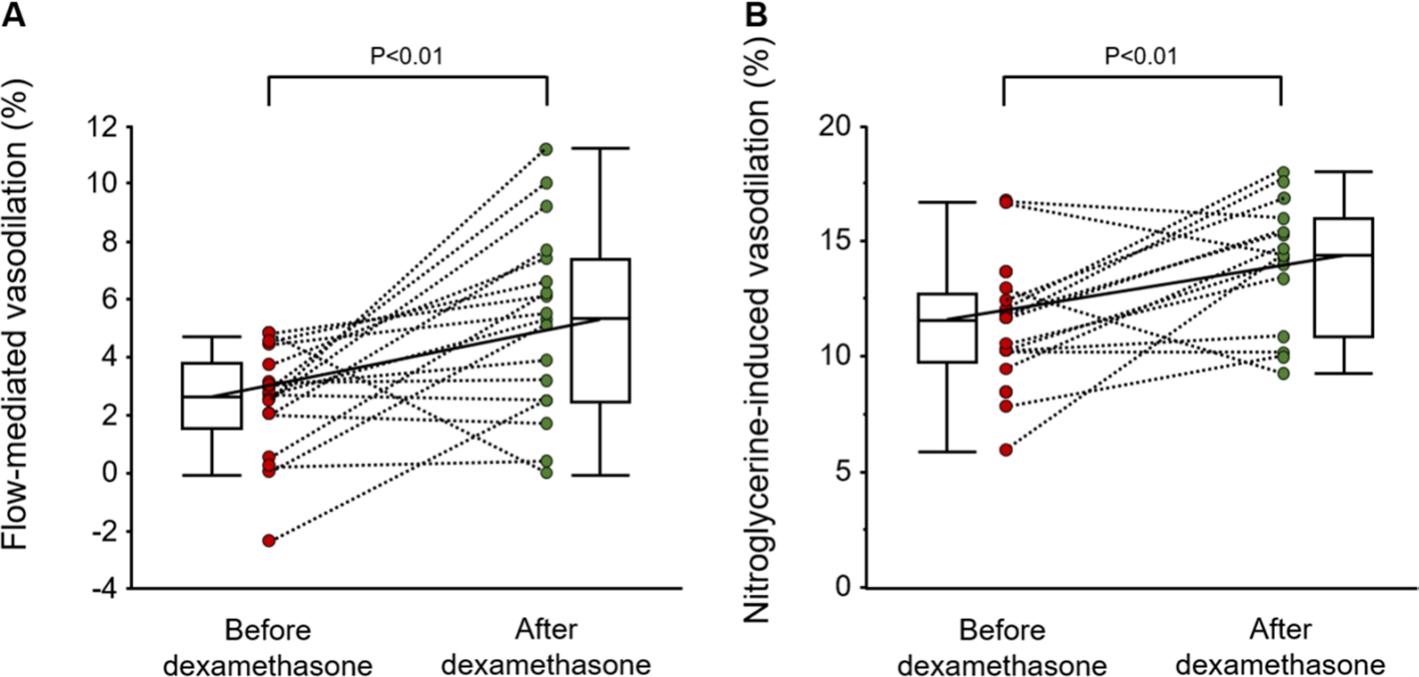
Dot plot graphs show flow-mediated vasodilation (**A**) and nitroglycerine-induced vasodilation (**B**) in patients with nonfunctioning adrenal adenomas before and after overnight 1 mg dexamethasone.

Overnight 1 mg dexamethasone decreased the morning plasma level of adrenocorticotropic hormone (ACTH) from 18.9 ± 10.1 to 2.1 ± 2.0 pg/mL (P < 0.01) and decreased morning plasma level of cortisol from 11.4 ± 3.9 to 1.1 ± 0.3 μg/dL (P < 0.01) but did not significantly alter blood pressure or heart rate (Table 3). Overnight 1 mg dexamethasone increased FMD from 2.4 ± 1.9% to 5.3 ± 3.2% (P < 0.01) and increased NID from 12.1 ± 4.2% to 14.0 ± 2.8% (P < 0.01) (Fig. 1).

**Table 3:** Effects of Overnight 1 mg Dexamethasone Suppression Test on Vascular Function and Variables in Patients with Nonfunctioning Adrenal Adenomas.

Variables	Before dexamethasone	After dexamethasone	P value
Systolic blood pressure, mmHg	134 ± 15	135 ± 18	0.52
Diastolic blood pressure, mmHg	79 ± 11	80 ± 11	0.90
Heart rate, bpm	74 ± 12	76 ± 15	0.45
Morning ACTH, pg/mL	18.9 ± 10.1	2.1 ± 2.0	< 0.01
Morning cortisol, μg/dL	11.4 ± 3.9	1.1 ± 0.3	< 0.01

*ACTH* adrenocorticotropic hormone.

Results are presented as means ± SD for continuous variables.

Changes in FMD after 1 mg dexamethasone were inversely correlated with changes in the morning plasma levels of cortisol (ρ = − 0.34, P = 0.04) (Supplemental Fig. S1A). There were no significant relationships between changes in FMD and changes in morning plasma levels of ACTH, blood pressure, and heart rate (Supplemental Fig. S1B–E). There were also no significant relationships between changes in NID and changes in morning plasma levels of cortisol, morning plasma levels of ACTH, blood pressure, and heart rate (Supplemental Fig. S1F–J).

## Discussion

In the present study, we demonstrated that there were no significant differences in FMD and NID between control patients with hypertension and/or DM and patients with NFA who had hypertension and/or DM even after adjustment for cardiovascular risk factors. Overnight 1 mg dexamethasone increased FMD and NID in patients with NFA. Increases in FMD after overnight 1 mg dexamethasone were inversely correlated with changes in morning plasma levels of cortisol. This is the first study to assess the impacts of overnight 1 mg dexamethasone on vascular function.

The prevalences of hypertension and DM are higher in patients with NFA than in healthy subjects. Androulakis et al.^6^ showed that endothelial function was impaired in patients with NFA who had no hypertension, DM, and/or dyslipidemia compared with that in healthy subjects. However, there is no information on the relationship between circulating levels of cortisol and vascular function in patients with NFA who have hypertension and/or DM. In the present study, there were no significant differences in vascular function between control patients with hypertension and/or DM and patients with NFA who had hypertension and/or DM even after adjustment for cardiovascular risk factors. These results supported the results of a previous study showing that the incidence of cardiovascular events in patients with NFA was comparable to that in patients without adrenal adenomas^4^. Sereg et al.^15^ showed that adrenalectomy in patients with NFA did not result in a decrease in cardiovascular events. Dalmazi et al.^2^ showed that the incidence of cardiovascular events was lower in patients with NFA than in patients with mild hypercortisolism, indicating that there might be little impact of NFA on cardiovascular events. In addition, in the present study, almost all of the patients with NFA were being treated with antihypertensive drugs and/or antidiabetic drugs. Pharmacological treatment for hypertension and/or DM in patients with NFA who have hypertension and/or DM may improve atherosclerosis and prevent cardiovascular events.

It is well established that there is a relationship of chronic glucocorticoid excess, including Cushing’s syndrome, mild autonomous cortisol secretion and iatrogenic Cushing’s syndrome, with increased incidence of cardiovascular disease^2,16–18^. Several studies have shown that patients who received more than 7.5 mg of prednisolone for more than three months were at an elevated risk of cardiovascular disease^8,9^. In addition, use of cortisol at a dose of 20 mg for five days has been shown to inhibit cholinergic vasodilation in healthy subjects^10^. On the other hand, Dover et al. showed that short-term administration of 2.8 mg hydrocortisone did not have acute effects on endothelial function in healthy subjects^19^. Brotman et al. showed that treatment with 3 mg dexamethasone twice daily for five days decreased NID, whereas there was no significant difference in FMD before and after treatment with 3 mg dexamethasone in healthy subjects^20^. In the present study, overnight 1 mg dexamethasone increased FMD and NID in patients with NFA. The discrepancy between the results of previous studies and the results of our study regarding the impacts of dexamethasone on FMD may be due to differences in the amounts and durations of dexamethasone administration.

Glucocorticoids can impact vascular reactivity by regulating either vasoconstriction or vasodilation. In the present study, changes in FMD after 1 mg dexamethasone inversely correlated with changes in morning plasma levels of cortisol, whereas there were no significant relationships between changes in FMD and changes in morning plasma levels of ACTH, blood pressure, and heart rate. These findings suggest that glucocorticoids directly affect vasodilation. Corticosteroids rapidly activate endothelial nitric oxide synthase via a non-transcriptional mechanism involving the glucocorticoid receptor and the PI3K/Akt pathway, leading to increased cerebral blood flow, reduced cerebral infarct size, and diminished vascular inflammation^21,22^. On the other hand, glucocorticoids regulate vasoconstrictors such as endothelin-1 and angiotensin-II, promoting vasoconstriction through an increase in endothelin-1 production and activation of angiotensin-II signaling, with notable impacts on atherosclerosis and endothelial cell dysfunction^23,24^. Glucocorticoids can amplify vasoconstriction by enhancing the expression of angiotensin-converting enzyme and angiotensin-II type I receptors, hence boosting intracellular signaling that triggers vessel contraction^25,26^. Glucocorticoids are both vasodilators and vasoconstrictors, whereas the observed impacts of overnight 1 mg dexamethasone (decrease in circulating levels of cortisol) may primarily be attributed to vasodilation.

This study has some limitations. First, the present study included a limited sample size of patients with NFA. However, overnight 1 mg dexamethasone improved vascular function in patients with NFA, even though the number of patients was small, and the sample size is statistically adequate to detect a 2.0% difference before and after administration of 1 mg dexamethasone. Further study is required to validate these findings in larger clinical trials. Second, in the present study, the overnight 1 mg dexamethasone suppression test was only performed for patients with NFA. It is unclear whether similar results apply to healthy subjects or patients without NFA. However, there were no significant differences in vascular function between control patients and patients with NFA. Therefore, similar results may apply to patients without NFA. Future studies are needed to confirm these findings in healthy subjects or patients without NFA.

## Conclusions

There were no significant differences in vascular function between control patients with hypertension and/or DM and patients with NFA who had hypertension and/or DM even after adjustment for cardiovascular risk factors. In patients with NFA, administration of overnight 1 mg dexamethasone is associated with increases in FMD and NID. Acute decreases in circulating levels of cortisol under the condition of normal adrenal function may cause acute improvements in FMD and NID.

## Methods

### Study protocol 1: vascular function in patients without adrenal incidentalomas and patients with NFA

This study was a single-center and prospective cohort study. Between August 2007 and August 2022, a total of 2657 subjects were recruited for vascular function measurements from individuals who attended the outpatient clinic at Hiroshima University Hospital. Of the 2657 subjects, 1105 subjects without computed tomography scans, 790 subjects with secondary hypertension, 399 subjects with cancer, four subjects with severe renal disease, 11 subjects with adrenal incidentalomas who did not receive detailed examinations, and 55 subjects without hypertension and/or DM were excluded. Finally, 22 patients with NFA who had hypertension and/or DM and 272 patients without adrenal incidentalomas who had hypertension and/or DM were enrolled in this study (Supplemental Figure S2).

Subclinical Cushing’s syndrome was defined according to the report of the guidelines for diagnostic criteria of adrenal subclinical Cushing’s syndrome: the Japan Endocrine Society 2018. Briefly, the criteria for diagnosis of adrenal subclinical Cushing’s syndrome include the presence of an adrenal mass, lack of characteristic features of Cushing’s syndrome, and normal basal serum cortisol levels. In addition, plasma levels of cortisol after a 1 mg dexamethasone suppression test were used to identify nonfunctioning adrenal incidentalomas (< 1.8 μg/dL), intermediate phenotype adrenal incidentalomas (1.8–5.0 μg/dL), and subclinical Cushing’s syndrome (≥ 5 μg/dL). Hypertension was defined as systolic blood pressure of more than140 mm Hg and/or diastolic blood pressure of more than 90 mm Hg measured in a sitting position on at least three different occasions in the outpatient clinic of Hiroshima University Hospital. Secondary hypertensive patients were excluded from the study as previously reported^27^.

Subjects fasted overnight for at least 12 h before the study. The subjects were kept in the supine position in a quiet, dark, air-conditioned room (constant temperature of 22 °C–25 °C) throughout the study. FMD, and NID were measured after maintaining the supine position for thirty minutes. The observers were blinded to the purposes of the study and the clinical status of the subjects. All methods were carried out in accordance with the Declaration of Helsinki, and relevant guidelines and regulations. The Ethics Review Board of Hiroshima University approved the study protocol. All participants in the study provided written informed consent.

### Study protocol 2: impacts of overnight 1 mg dexamethasone on vascular function in patients with NFA

Between November 2020 and August 2022, a total of 28 consecutive patients with adrenal incidentalomas were recruited for vascular function measurements from patients who attended the outpatient clinic at Hiroshima University Hospital. Ten of the 28 patients with adrenal incidentalomas, including five patients with subclinical Cushing’s syndrome three patients with Cushing’s syndrome, and two patients with primary aldosteronism, were excluded. Finally, 18 patients with NFA were enrolled in this study. FMD and NID were measured in the morning before and after administration of 1 mg of dexamethasone at 2300 h as a dexamethasone suppression test for all patients.

### Measurements of FMD and NID

FMD was measured in vascular response to reactive hyperemia in the brachial artery as endothelium-dependent vasodilation. A high-resolution linear artery transducer was coupled to computer-assisted analysis software (UNEXEF18G, UNEX Co, Nagoya, Japan) that used an automated edge detection system for measurement of brachial artery diameter^28^. NID was measured in vascular response to nitroglycerine as endothelium-independent vasodilation, as previously reported^28^. Additional details are available in the online-only Data Supplement.

### Statistical analysis

Results are summarized as means ± SD for continuous variables and percentages for categorical variables. Statistical significance was a probability value of < 0.05. The comparison of continuous variables was conducted using ANOVA with Tukey’s post hoc test. The comparison of categorical variables between groups was conducted using a chi-square test. The associations between variables were assessed by using Spearman’s correlation coefficients. Changes in parameters after administration of 1 mg dexamethasone were evaluated using the paired t-test. To create a matched cohort of patients with NFA who had hypertension and/or DM and patients without adrenal incidentalomas with hypertension and/or DM in protocol 1, a propensity score was computed for each patient by using logistic regression analysis to determine the probability of baseline clinical variables including age, sex, body mass index, hypertension, dyslipidemia, DM, current smokers, and precious coronary heart disease. To create a matched cohort of patients with NFA and patients without adrenal incidentalomas in protocol 2, a propensity score was computed for each patient by using logistic regression analysis to determine the probability of baseline clinical variables including age, sex, body mass index, hypertension, dyslipidemia, DM, current smokers and precious coronary heart disease. One-to-one propensity-score matching analyses were used to create matched pairs to investigate the associations of NFA with vascular function. With these propensity scores, two well-matched groups based on clinical characteristics were created with a caliper size specification (0.25 × SD of propensity score) for comparison of vascular function. Propensity-score matching analyses were used to reduce the effects of selection bias. To detect a 2.0% difference before and after administration of 1 mg dexamethasone with α of 0.05 and power 0.90, the total sample size requirement would exceed 14. We performed multiple imputations for incomplete data, since multiple imputations of missing data have been recommended to avoid potential bias in full case analysis due to missing values. The data were processed using JMP pro version 15 (SAS Institute. Cary, NC).

### Supplementary Information

Supplementary Information.

## Data Availability

The data presented in this study are available on request from the corresponding author.

## References

[1] Mantero F (2000). A survey on adrenal incidentaloma in Italy. Study Group on Adrenal Tumors of the Italian Society of Endocrinology. J. Clin. Endocrinol. Metab..

[2] Di Dalmazi G (2014). Cardiovascular events and mortality in patients with adrenal incidentalomas that are either non-secreting or associated with intermediate phenotype or subclinical Cushing's syndrome: A 15-year retrospective study. Lancet Diabetes Endocrinol..

[3] Patrova J, Mannheimer B, Lindh JD, Falhammar H (2023). Mortality in patients with nonfunctional adrenal tumors. JAMA Intern. Med..

[4] Lopez D (2016). "Nonfunctional" adrenal tumors and the risk for incident diabetes and cardiovascular outcomes: A cohort study. Ann. Intern. Med..

[5] Favero V (2023). The degree of cortisol secretion is associated with diabetes mellitus and hypertension in patients with nonfunctioning adrenal tumors. Cardiovasc. Diabetol..

[6] Androulakis II (2014). Patients with apparently nonfunctioning adrenal incidentalomas may be at increased cardiovascular risk due to excessive cortisol secretion. J. Clin. Endocrinol. Metab..

[7] Walker BR (2007). Glucocorticoids and cardiovascular disease. Eur. J. Endocrinol..

[8] Wei L, MacDonald TM, Walker BR (2004). Taking glucocorticoids by prescription is associated with subsequent cardiovascular disease. Ann. Intern. Med..

[9] Souverein PC (2004). Use of oral glucocorticoids and risk of cardiovascular and cerebrovascular disease in a population based case-control study. Heart.

[10] Mangos GJ (2000). Cortisol inhibits cholinergic vasodilation in the human forearm. Am. J. Hypertens..

[11] Ross R (1999). Atherosclerosis–an inflammatory disease. N. Engl. J. Med..

[12] Higashi Y, Noma K, Yoshizumi M, Kihara Y (2009). Endothelial function and oxidative stress in cardiovascular diseases. Circ. J..

[13] Celermajer DS (1992). Non-invasive detection of endothelial dysfunction in children and adults at risk of atherosclerosis. Lancet (London, England).

[14] Corretti MC (2002). Guidelines for the ultrasound assessment of endothelial-dependent flow-mediated vasodilation of the brachial artery: A report of the International Brachial Artery Reactivity Task Force. J. Am. Coll. Cardiol..

[15] Sereg M (2009). Atherosclerotic risk factors and complications in patients with non-functioning adrenal adenomas treated with or without adrenalectomy: A long-term follow-up study. Eur. J. Endocrinol..

[16] Etxabe J, Vazquez JA (1994). Morbidity and mortality in Cushing's disease: An epidemiological approach. Clin. Endocrinol..

[17] Fardet L, Petersen I, Nazareth I (2012). Risk of cardiovascular events in people prescribed glucocorticoids with iatrogenic Cushing's syndrome: cohort study. BMJ.

[18] Fassnacht M (2023). European Society of Endocrinology clinical practice guidelines on the management of adrenal incidentalomas, in collaboration with the European Network for the Study of Adrenal Tumors. Eur. J. Endocrinol..

[19] Dover AR, Hadoke PW, Walker BR, Newby DE (2007). Acute effects of glucocorticoids on endothelial fibrinolytic and vasodilator function in humans. J. Cardiovasc. Pharmacol..

[20] Brotman DJ (2005). Effects of short-term glucocorticoids on cardiovascular biomarkers. J. Clin. Endocrinol. Metabol..

[21] Limbourg FP (2002). Rapid nontranscriptional activation of endothelial nitric oxide synthase mediates increased cerebral blood flow and stroke protection by corticosteroids. J. Clin. Investing..

[22] Hafezi-Moghadam A (2002). Acute cardiovascular protective effects of corticosteroids are mediated by non-transcriptional activation of endothelial nitric oxide synthase. Nat. Med..

[23] Sutton G, Pugh D, Dhaun N (2019). Developments in the role of endothelin-1 in atherosclerosis: A potential therapeutic target?. Am. J. Hypertens..

[24] Danser AH, Admiraal PJ, Derkx FH, Schalekamp MA (1998). Angiotensin I-to-II conversion in the human renal vascular bed. J. Hypertens..

[25] Coulet F (2001). Endothelium-independent conversion of angiotensin I by vascular smooth muscle cells. Cell Tissue Res..

[26] Sato A (1994). Increased expression of vascular angiotensin II type 1A receptor gene in glucocorticoid-induced hypertension. J. Hypertens..

[27] Kishimoto S (2020). A comparison of adrenalectomy and eplerenone on vascular function in patients with aldosterone-producing adenoma. J. Clin. Endocrinol. Metab..

[28] Maruhashi T (2013). Nitroglycerine-induced vasodilation for assessment of vascular function: A comparison with flow-mediated vasodilation. Arterioscler. Thromb. Vascul. Biol..

