# Supplementary material for: Impact of overnight 1 mg dexamethasone on vascular function in patients with nonfunctioning adrenal adenomas
Source: Sci Rep. 2023 Nov 28;13:20975. doi: 10.1038/s41598-023-48295-y (PMC10684497; doi:10.1038/s41598-023-48295-y)
Supplement: Supplementary file 1 — Supplementary Information. [file 41598_2023_48295_MOESM1_ESM.docx]

**Online Supplement**

**Impact of Overnight 1 mg Dexamethasone on Vascular Function in Patients with Nonfunctioning Adrenal Adenomas**

Running title: Dexamethasone and endothelial function

Shinji Kishimoto, MD, PhD;^1^ Tatsuya Maruhashi, MD, PhD;^1^ Masato Kajikawa, MD, PhD;^2^ Aya Mizobuchi, MS;^1^ Takayuki Yamaji, MD, PhD;^3^ Takahiro Harada, MD, PhD;^4^ Yukiko Nakano, MD, PhD;^5^ Chikara Goto, PhD;^6^ Farina Mohamad Yusoff, MD, PhD;^1^ Ayumu Nakashima, MD, PhD;^7^ Yukihito Higashi, MD, PhD, FAHA^1,2^

^1^Department of Regenerative Medicine, Division of Radiation Medical Science, Research Institute for Radiation Biology and Medicine, Hiroshima University, Hiroshima, Japan

^2^Division of Regeneration and Medicine, Medical Center for Translational and Clinical Research, Hiroshima University Hospital, Hiroshima, Japan

^3^Center for Radiation Disaster Medical Science, Research Institute for Radiation Biology and Medicine, Hiroshima University, Hiroshima, Japan

^4^Center for Cause of Death Investigation Research, Graduate School of Biomedical and Health Sciences, Hiroshima University, Hiroshima, Japan

^5^Department of Cardiovascular Medicine, Graduate School of Biomedical and Health Sciences, Hiroshima University, Hiroshima, Japan

^6^Dpartment of Rehabilitation, Faculty of General Rehabilitation, Hiroshima International University, Hiroshima, Japan

^7^Department of Nephrology, Graduate School of Medicine, University of Yamanashi, Yamanashi, Japan

Address for correspondence: Yukihito Higashi, MD, PhD, FAHA

Department of Regenerative Medicine, Division of Radiation Medical Science,

Research Institute for Radiation Biology and Medicine (RIRBM), Hiroshima University

1-2-3 Kasumi, Minami-ku, Hiroshima 734-8551, Japan

Phone: +81-82-257-5831 Fax: +81-82-257-5831

E-mail: yhigashi@hiroshima-u.ac.jp

**Methods**

**Measurements of FMD and NID**

Vascular response to reactive hyperemia in the brachial artery was used for assessment of endothelium-dependent FMD. A high-resolution linear artery transducer was coupled to computer-assisted analysis software (UNEXEF18G, UNEX Co, Nagoya, Japan) that used an automated edge detection system for measurement of brachial artery diameter.^1^ A blood pressure cuff was placed around the forearm. The brachial artery was scanned longitudinally 5-10 cm above the elbow. When the clearest B-mode image of the anterior and posterior intimal interfaces between the lumen and vessel wall was obtained, the transducer was held at the same point throughout the scan by a special probe holder (UNEX Co) to ensure consistency of the image. Depth and gain setting were set to optimize the images of the arterial lumen wall interface. When the tracking gate was placed on the intima, the artery diameter was automatically tracked, and the waveform of diameter changes over the cardiac cycle was displayed in real time using the FMD mode of the tracking system. This allowed the ultrasound images to be optimized at the start of the scan and the transducer position to be adjusted immediately for optimal tracking performance throughout the scan. Pulsed Doppler flow was assessed at baseline and during peak hyperemic flow, which was confirmed to occur within 15 seconds after cuff deflation. Blood flow velocity was calculated from the color Doppler data and was displayed as a waveform in real time. The baseline longitudinal image of the artery was acquired for 30 seconds, and then the blood pressure cuff was inflated to 50 mm Hg above systolic pressure for 5 minutes. The longitudinal image of the artery was recorded continuously until 5 minutes after cuff deflation. Pulsed Doppler velocity signals were obtained for 20 seconds at baseline and for 10 seconds immediately after cuff deflation. Changes in brachial artery diameter were immediately expressed as percentage change relative to the vessel diameter before cuff inflation. FMD was automatically calculated as the percentage change in peak vessel diameter from the baseline value. Percentage of FMD [(Peak diameter - Baseline diameter)/Baseline diameter] was used for analysis. Blood flow volume was calculated by multiplying the Doppler flow velocity (corrected for the angle) by heart rate and vessel cross-sectional area (-r2). Reactive hyperemia was calculated as the maximum percentage increase in flow after cuff deflation compared with baseline flow.

The response to nitroglycerine was used for assessment of endothelium-independent vasodilation. NID was measured as described previously.^1^ Briefly, after acquiring baseline rest images for 30 seconds, a sublingual tablet (75 μg nitroglycerine) was given, and images of the artery was recorded continuously until the dilation reached a plateau after administration of nitroglycerine. Subjects who had received nitrate treatment and subjects in whom the sublingually administered nitroglycerine tablet was not dissolved during the measurement were excluded from this study. NID was automatically calculated as a percent change in peak vessel diameter from the baseline value. Percentage of NID [(Peak diameter - Baseline diameter)/Baseline diameter] was used for analysis. Inter- and intra-coefficients of variation for the brachial artery diameter were 1.6% and 1.4%, respectively, in our laboratory.

**References**

1 Maruhashi, T. et al. Nitroglycerine-induced vasodilation for assessment of vascular function: a comparison with flow-mediated vasodilation. Arteriosclerosis, thrombosis, and vascular biology 33, 1401-1408, doi:10.1161/atvbaha.112.300934 (2013).

**Supplemental Table S1.** Clinical Characteristics of the Subjects in Protocol 1

| Variables | Control  (n=272) | Nonfunctioning adrenal adenomas (n=22) | P value |
| --- | --- | --- | --- |
| Age, year | 68±10 | 64±10 | 0.07 |
| Sex, men/women | 179/93 | 18/4 | 0.11 |
| Body mass index, kg/m^2^ | 24.3±4.0 | 24.0±6.8 | 0.76 |
| Systolic blood pressure, mmHg | 133±18 | 135±17 | 0.68 |
| Diastolic blood pressure, mmHg | 78±11 | 80±13 | 0.36 |
| Heart rate, bpm | 71±12 | 76±13 | 0.05 |
| Total cholesterol, mmol/L | 4.73±0.98 | 4.84±0.75 | 0.62 |
| Triglycerides, mmol/L | 1.51±0.88 | 1.82±1.47 | 0.14 |
| High-density lipoprotein cholesterol, mmol/L | 1.55±0.44 | 1.42±0.52 | 0.23 |
| Low-density lipoprotein cholesterol, mmol/L | 2.66±0.78 | 2.79±0.67 | 0.45 |
| Glucose, mmol/L | 6.88±2.44 | 6.16±1.22 | 0.17 |
| Hemoglobin A1c, % | 6.3±0.9 | 6.1±0.8 | 0.35 |
| Blood urea nitrogen, mmol/L | 5.71±2.14 | 5.36±1.43 | 0.56 |
| Creatinine, μmol/L | 73.37±22.10 | 73.37±21.22 | 0.97 |
| Current smoker, n (%) | 34 (12.5) | 6 (27.3) | 0.08 |
| Medical history, n (%) |  |  |  |
| Hypertension | 246 (90.4) | 20 (90.4) | 0.94 |
| Dyslipidemia | 174 (64.0) | 13 (59.1) | 0.62 |
| Diabetes mellitus | 136 (50.0) | 7 (31.8) | 0.09 |
| Previous coronary heart disease | 72 (26.5) | 0 (0.0) | <0.01 |
| Previous stroke | 25 (9.2) | 3 (13.6) | 0.55 |
| Medication, n (%) |  |  |  |
| Calcium channel blockers | 141 (51.8) | 12 (54.6) | 0.81 |
| Angiotensin-converting enzyme inhibitors | 14 (5.1) | 0 (0.0) | 0.14 |
| Angiotensin II receptor blockers | 152 (55.9) | 9 (40.9) | 0.18 |
| Mineralocorticoid receptor blockers | 26 (9.6) | 0 (0.0) | 0.04 |
| Beta-blockers | 63 (23.2) | 1 (4.6) | 0.02 |
| Alpha-blockers | 14 (5.1) | 1 (4.6) | 0.90 |
| Statins | 121 (44.5) | 6 (27.3) | 0.10 |
| Nitrates | 21 (7.7) | 0 (0.0) | 0.07 |
| Medically treated diabetes mellitus |  |  |  |
| Any | 91 (33.5) | 2 (9.1) | <0.01 |
| Insulin-dependent | 14 (5.1) | 0 (0.0) | 0.13 |
| Flow-mediated vasodilation, % | 3.4±2.8 | 2.9±1.9 | 0.46 |
| Nitroglycerine-induced vasodilation, % | 11.5±5.7 | 11.4±4.3 | 0.96 |

Results are presented as means±SD for continuous variables and percentages for categorical variables.

**Supplemental Table S2.** Clinical Characteristics of the Subjects in Protocol 2

| Variables | Control  (n=320) | Nonfunctioning adrenal adenomas (n=18) | P value |
| --- | --- | --- | --- |
| Age, year | 66±12 | 65±10 | 0.58 |
| Sex, men/women | 214/107 | 14/4 | 0.31 |
| Body mass index, kg/m^2^ | 23.9±4.4 | 25.8±2.6 | 0.07 |
| Systolic blood pressure, mmHg | 132±18 | 134±15 | 0.66 |
| Diastolic blood pressure, mmHg | 77±11 | 79±11 | 0.37 |
| Heart rate, bpm | 72±13 | 74±12 | 0.53 |
| Total cholesterol, mmol/L | 4.78±0.96 | 4.84±0.72 | 0.79 |
| Triglycerides, mmol/L | 1.60±1.12 | 1.93±1.61 | 0.24 |
| High-density lipoprotein cholesterol, mmol/L | 1.53±0.47 | 1.32±0.49 | 0.06 |
| Low-density lipoprotein cholesterol, mmol/L | 2.69±0.78 | 2.84±0.62 | 0.53 |
| Glucose, mmol/L | 6.77±2.33 | 6.11±1.11 | 0.22 |
| Hemoglobin A1c, % | 6.2±0.9 | 5.9±0.8 | 0.16 |
| Blood urea nitrogen, mmol/L | 5.71±2.14 | 5.36±1.79 | 0.56 |
| Creatinine, μmol/L | 73.37±24.75 | 76.02±22.10 | 0.64 |
| Current smoker, n (%) | 44 (13.8) | 3 (16.7) | 0.75 |
| Medical history, n (%) |  |  |  |
| Hypertension | 246 (76.9) | 15 (83.3) | 0.57 |
| Dyslipidemia | 197 (61.6) | 11 (61.1) | 0.90 |
| Diabetes mellitus | 136 (42.5) | 4 (22.2) | 0.07 |
| Previous coronary heart disease | 76 (23.8) | 0 (0.0) | <0.01 |
| Previous stroke | 29 (9.1) | 3 (16.7) | 0.36 |
| Medication, n (%) |  |  |  |
| Calcium channel blockers | 146 (45.6) | 8 (44.4) | 0.88 |
| Angiotensin-converting enzyme inhibitors | 14 (4.4) | 0 (0.0) | 0.21 |
| Angiotensin II receptor blockers | 152 (47.5) | 6 (33.3) | 0.21 |
| Mineralocorticoid receptor blockers | 26 (8.1) | 0 (0.0) | 0.09 |
| Beta-blockers | 66 (20.6) | 1 (5.6) | 0.28 |
| Alpha-blockers | 15 (4.7) | 1 (5.6) | 0.88 |
| Statins | 133 (41.6) | 5 (27.8) | 0.22 |
| Nitrates | 23 (7.2) | 0 (0.0) | 0.10 |
| Medically treated diabetes mellitus |  |  |  |
| Any | 91 (28.4) | 1 (5.6) | 0.01 |
| Insulin-dependent | 14 (4.4) | 0 (0.0) | 0.21 |
| Flow-mediated vasodilation, % | 3.4±2.8 | 2.4±1.9 | 0.16 |
| Nitroglycerine-induced vasodilation, % | 11.7±5.8 | 12.1±4.2 | 0.78 |

Results are presented as means±SD for continuous variables and percentages for categorical variables.

**Supplemental Figure S1**


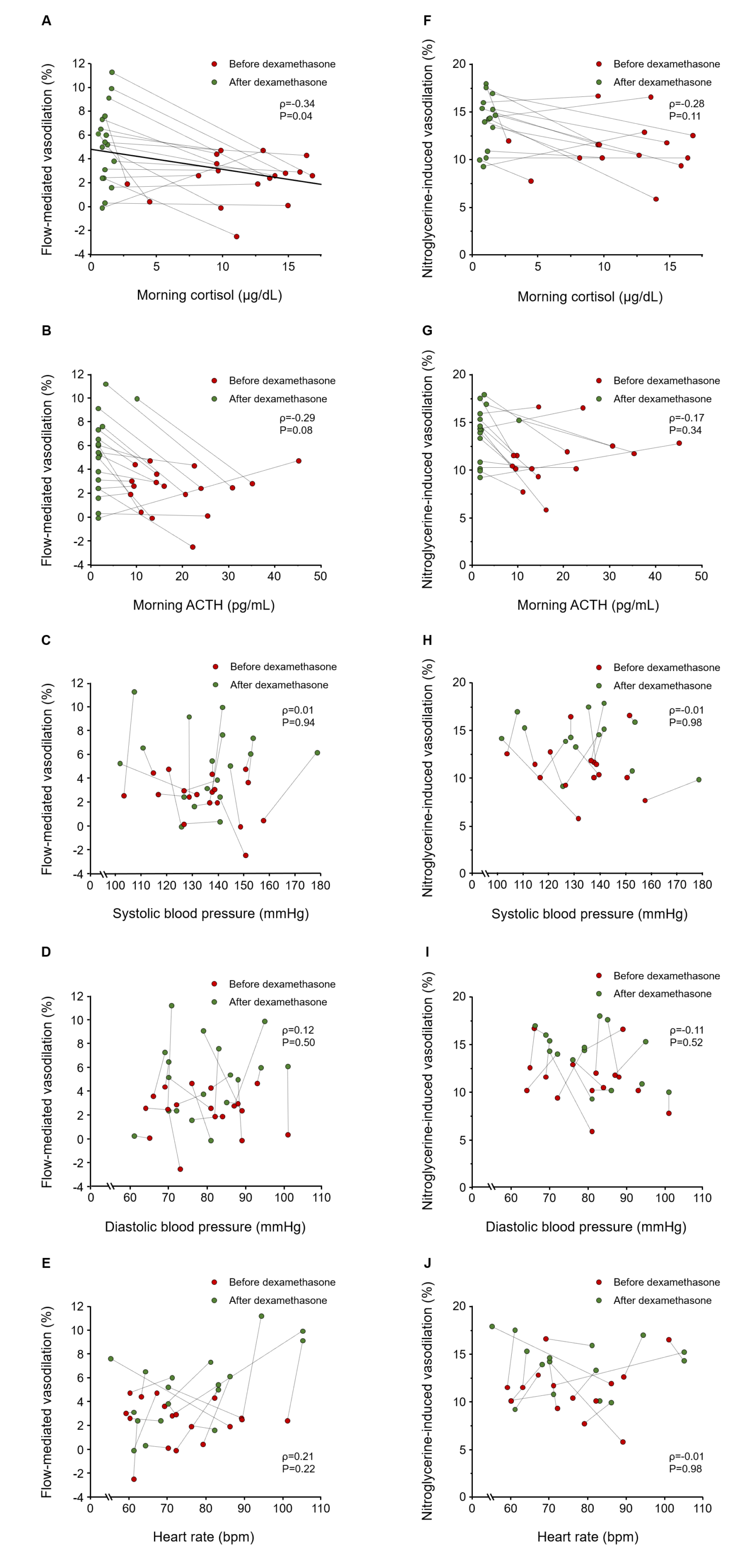


Supplemental Figure S1 Legend:

Relationships between changes in flow-mediated vasodilation and changes in morning cortisol (**A**), morning adrenocorticotropic hormone (ACTH) (**B**), systolic blood pressure (**C**), diastolic blood pressure (**D**), and heart rate (**E**) and relationships between changes in nitroglycerine-induced vasodilation and changes in morning cortisol (**F**), morning ACTH (**G**), systolic blood pressure (**H**), diastolic blood pressure (**I**) and heart rate (**J**) in patients with non-functioning adrenal incidentalomas.

**Supplemental Figure S2**

**
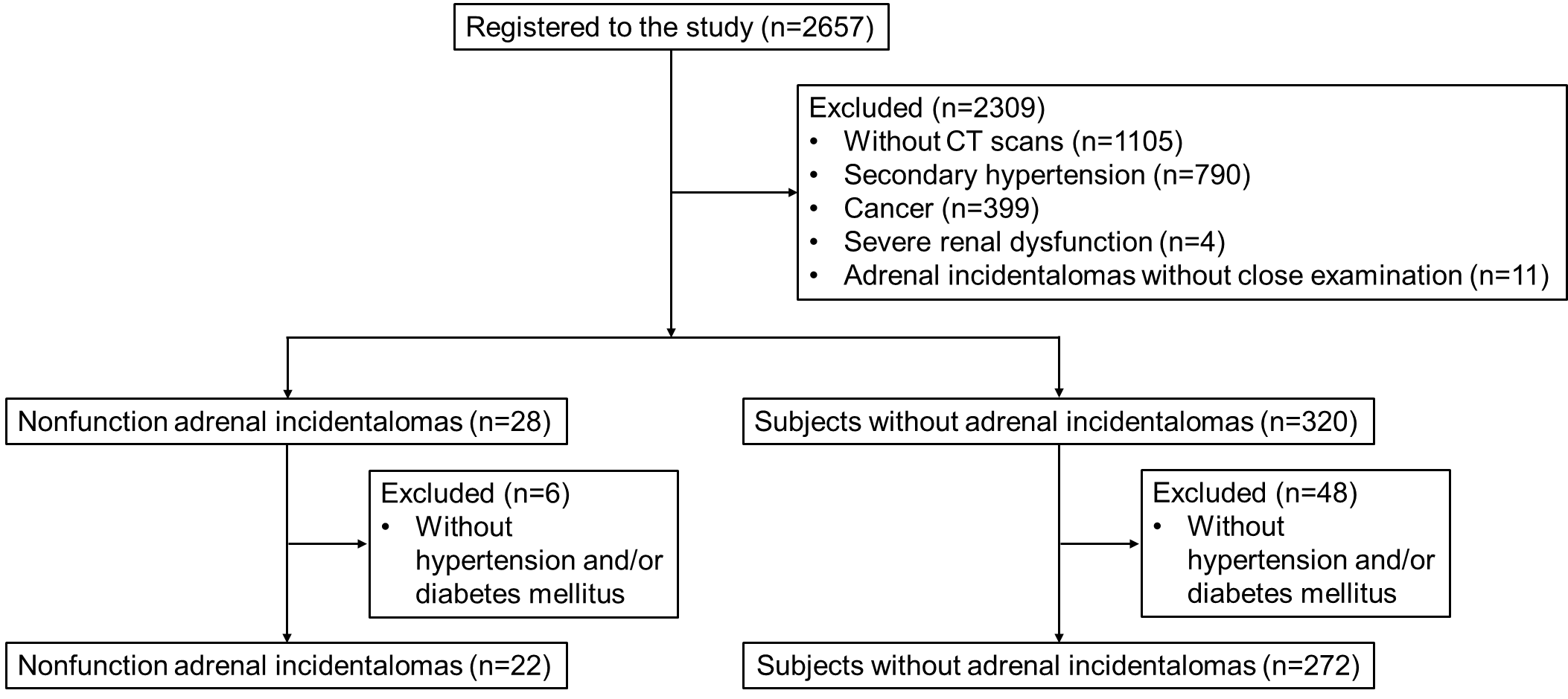
**

Supplemental Figure S2 Legend:

Flow diagram of participants in this study
